# Autoimmune Neutropenia and Immune-Dysregulation in a Patient Carrying a *TINF2* Variant

**DOI:** 10.3390/ijms232314535

**Published:** 2022-11-22

**Authors:** Benedetta Chianucci, Alice Grossi, Gianluca Dell’Orso, Elena Palmisani, Marina Lanciotti, Paola Terranova, Filomena Pierri, Michela Lupia, Luca Arcuri, Marica Laurino, Isabella Ceccherini, Fabian Beier, Carlo Dufour, Francesca Fioredda, Maurizio Miano

**Affiliations:** 1 Haematology Unit, IRCCS Istituto Giannina Gaslini, 16147 Genoa, Italy; 2 Genetics and Genomics of Rare Diseases, IRCCS Istituto Giannina Gaslini, 16147 Genoa, Italy; 3 Hematopoietic Stem Cell Transplant Unit, Department of Hematology and Oncology, IRCCS Istituto Giannina Gaslini, 16147 Genoa, Italy; 4 Hematology and Cellular Therapy Unit, IRCCS Ospedale Policlinico San Martino, 16132 Genoa, Italy; 5 Department of Hematology, Oncology, Hemostaseology, Stem Cell Transplantation, Medical Faculty, RWTH Aachen University, 52047 Aachen, Germany; 6 Center for Integrated Oncology Aachen Bonn Cologne Düsseldorf (CIO ABCD), 53127 Bonn, Germany

**Keywords:** bone marrow failure (BMF), immune-dysregulation, next generation sequencing (NGS), autoimmune neutropenia (AIN), dyskeratosis congenita (DC), telomeropathy

## Abstract

In recent years, the knowledge about the immune-mediated impairment of bone marrow precursors in immune-dysregulation and autoimmune disorders has increased. In addition, immune-dysregulation, secondary to marrow failure, has been reported as being, in some cases, the most evident and early sign of the disease and making the diagnosis of both groups of disorders challenging. Dyskeratosis congenita is a disorder characterized by premature telomere erosion, typically showing marrow failure, nail dystrophy and leukoplakia, although incomplete genetic penetrance and phenotypes with immune-dysregulation features have been described. We report on a previously healthy 17-year-old girl, with a cousin successfully treated for acute lymphoblastic leukemia, who presented with leukopenia and neutropenia. The diagnostic work-up showed positive anti-neutrophil antibodies, leading to the diagnosis of autoimmune neutropenia, a slightly low NK count and high TCR-αβ+-double-negative T-cells. A next-generation sequencing (NGS) analysis showed the 734C>A variant on exon 6 of the *TINF2* gene, leading to the p.Ser245Tyr. The telomere length was short on the lymphocytes and granulocytes, suggesting the diagnosis of an atypical telomeropathy showing with immune-dysregulation. This case underlines the importance of an accurate diagnostic work-up of patients with immune-dysregulation, who should undergo NGS or whole exome sequencing to identify specific disorders that deserve targeted follow-up and treatment.

## 1. Introduction

Bone marrow failure (BMF) is a rare but life-threatening condition due to defective hematopoiesis of the bone marrow, resulting in cytopenia/pancytopenia. It can be inherited or acquired: the inherited forms are more frequent early in life [1,2] and are often associated with physical abnormalities and a predisposition to malignancy [1,3]. The diagnostic work-up of BMF has been evolving over the years, due to the increasing knowledge of the interplay between the immune system and bone marrow function, as suggested by the involvement of bone marrow precursors in the setting of immune-dysregulation and autoimmune disorders [4,5] and by the impairment of the immune system as an early sign of BMF [6,7,8,9,10,11,12].

Dyskeratosis congenita (DC) is a rare telomere biology disorder, characterized by the presence of very short telomeres due to premature erosion. It typically presents with BMF, associated with the clinical triad including abnormal skin pigmentation, nail dystrophy and leukoplakia [13], although other organs are usually involved. Pancytopenia, malignancies and pulmonary fibrosis are often present. Incomplete genetic penetrance, disease anticipation and atypical phenotypes with immune-dysregulation features have been consistently reported [7,10,12]. Telomere-mediated T-cell immune-dysregulation can be the first presentation of marrow failure that can precede clinically significant cytopenia, possibly leading to premature morbidity and mortality if under/misdiagnosed and under/mistreated [7,10]. Even if about 20% of patients with DC can show autoimmune or lymphoproliferative symptoms [12], few data are reported on this specific aspect.

Autoimmune neutropenia (AIN) is the most clinically relevant form of acquired neutropenia in children. It is usually a benign condition, caused by antibodies against human neutrophil antigens, presenting in early infancy and usually self-limiting in about 2 years [14]. Despite that, cases with later onset and/or persisting disease or additional cell-lineage involvement are reported and need to undergo accurate immunological work-up for the possible diagnosis of secondary forms of AIN [15,16,17]. Herein, we describe a patient with isolated AIN who carried a variant of the *TINF2* gene and short telomere length.

## 2. Case Presentation

A previously healthy female patient with a family history of a paternal first cousin successfully treated for a B-cell acute lymphoblastic leukemia (ALL), at the age of 17, presented with isolated leucopenia and severe neutropenia (white blood count 2580/mmc, neutrophil 290/mmc) and underwent investigation in another hospital, which ruled out hematological neoplasms and myelodysplastic syndromes. No history of respiratory impairment was reported and no abnormalities were present at the physical examination; in particular, the skin, nails and tongue appeared normal. She was then referred to our center for an evaluation of the neutropenia.

**Figure 1:**
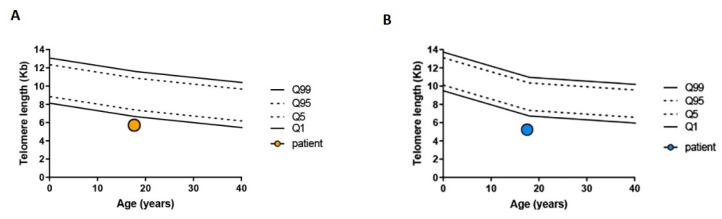
Telomere analysis of the patient measured with flow-FISH. (**A**) Telomere length of peripheral blood lymphocytes (orange circle). (**B**) Telomere length of peripheral blood granulocytes (blue circle). The 1 and 99 percentile are depicted as solid line. The 5 and 95 as dashed line.

The immunological diagnostic work-up showed normal T, B and CD4/CD8 ratios, a slightly low natural killer (NK) count, increased CD3+ TCR-γ/δ+ cells (17%) and high levels of TCR-αβ+-double-negative T-cells (3.9% of total lymphocytes). The immunoglobulin serum levels, IgG subclasses and responses to tetanus and diphtheria were normal. The serum anti-neutrophil antibodies, detected by the indirect Flow-Gift [18], were positive at the third evaluation, and the Vitamin B12 serum level was above the normal range (816 pg/mL, n.v. 191–663). Table 1 and Table 2 show the most significant laboratory tests performed in our center. According to the Consensus Guidelines on Diagnosis, from the Neutropenia Committee of the Marrow Failure Syndrome Group of the Italian Association of Pediatric Hematology/Oncology, based on the positivity of anti-neutrophil antibodies, the patient was diagnosed with AIN [19]. Based on these findings and the family history, a next-generation sequencing (NGS) analysis of 58 genes related to both immune-dysregulation syndromes and bone marrow failures was performed, whose details are reported by Grossi et al. [20]. A heterozygous variant was detected in the *TINF2* gene (NM_001099274.3), a gene associated with DC, already described in cases of aplastic anemia [21]. In particular, it is the c.734C>A variant, leading to the p.Ser245Tyr missense change, found with a 6.7E-4 frequency among (non-Finnish)-Europeans and predicted with effects ranging from likely pathogenic [21,22,23,24] to likely benign (https://varsome.com/variant/hg38/TINF2%20S245Y?annotation-mode=germline; accessed on 14 February 2022). Table 3 shows the coverage rate of all the genes included in the NGS panel. Interestingly, the patient’s cousin from the father’s side, who was affected by ALL and tested in remission, did not carry the same variant, which, instead, was inherited from the mother. The telomere length was then measured in two separate assays and was very short (<1%) in both the total lymphocytes and the granulocytes (Figure 1). No clinical signs of DC were present and the skin, nails and tongue appeared normal. The marrow aspiration was repeated and revealed an absence of atypical cells and normal precursor maturation. A Colony Forming Units Assay (CFU-A) was normal (Table 4). A trephine biopsy showed normal cellularity associated with reduced myelopoiesis and mild dyserythropoiesis; the search for immature myeloid elements performed by immunohistochemical CD34 staining was negative. The response to Granulocyte-Colony Stimulating Factor (G-CSF) was tested after stimulation at the dose of 300 µg and the neutrophil count rose from 280/mmc to 1260/mmc, 1090/mmc and 450/mmc after 3, 6 and 24 h, respectively. The patient’s mother had a normal blood cell count with no relevant clinical issues. In order to exclude further underlying genetic defects, Whole Exome Sequencing (WES) was also performed, and the result was negative. At the 4-year follow-up, the patient showed persistent severe neutropenia (the neutrophils count ranging 150–430/mmc, median 260/mmc) and suffered from several episodes of hydradenitis suppurativa, which was responsive to antibiotic therapy.

## 3. Discussion

This case report highlights that AIN can be an epiphenomenon of underlying disorders that may deserve further investigation. The classical and prevalent clinical phenotype of AIN is the presentation in the first years of life and spontaneous resolution within 24–36 months, with no relevant clinical issues. However, AIN cases are known to present later in life with no tendency to resolve, requiring deeper investigation in order to identify potential underlying diseases [16]. In this regard, in addition to the immunological screening, a genetic analysis represents a fundamental tool to conclude the diagnostic work-up of selected cases, since the well-known heterogeneous clinical phenotype and the incomplete penetrance of immune-dysregulation syndromes may lead to the wrong diagnosis. In this setting, NGS or WES should be offered to all patients [20,25]. In our case, the late onset of AIN and the family history induced us to perform, with a high priority, an NGS analysis of the genes involved in both immune-dysregulation syndromes and BMF. Rather than the expected variants of genes causing inborn errors of immunity (IEI), the sequencing results showed a rare heterozygous variant—inherited from the mother—with conflicting interpretations of its effect in the *TINF2* gene, which is known to cause telomere biology diseases. Moreover, since the variant was not found in her paternal cousin, who had been previously treated for a B-cell ALL with no specific molecular alterations, we were able to exclude any correlation between the two cases. Such an unexpected result was obtained thanks to the use of a wide NGS panel, including genes related to both IEI and BMF, intentionally designed in our center in order to identify atypical phenotypes in both groups of disorders [20]. This approach led us to describe a considerable number of BMF secondary to IEI [25], highlighting a significant clinical/pathogenic overlap between such disorders. Nonetheless, Allenspach et al. also described a case of DC that, for a long time in childhood, displayed an isolated antibody deficiency, leading to an initial diagnosis of common variable immunodeficiency (CVID) but that, over time, developed BMF [12], thus suggesting that marrow failure syndromes may have a long preceding phase characterized only by immune-dysregulation [6,7,8,9,10,11,12]. In our patient, the presence of anti-neutrophil antibodies and the response to G-CSF stimulation in the setting of normal marrow cellularity and the CFU assay suggests a prevalence of immune-mediated pathogenesis of neutropenia and still-preserved hematopoiesis, which, however, needs to be monitored over time. Of note, the co-existence of BMF and immune-dysregulation is documented to occur in other rare disorders, such as DNA-repair syndromes and deficiencies of *GATA2* or *ADA2* [26,27,28,29,30], thus reinforcing the concept that a wide diagnostic work-up can be appropriate in these cases. DC is characterized by a short telomere length leading to premature stem cell consumption. The clinical presentation of the disease is very heterogeneous, varying from mild to critical forms, depending on the gene variant. In about 60–70% of cases, anomalies in the telomere biology genes are associated, and shelterin complex proteins (TINF2) mutations are the cause of about 15% of DC. We recently described the VUS Ser245Tyr on the *TINF2* gene [21] in other patients with bone marrow failure in whom, unfortunately, we could not perform the telomere length analysis. Instead, in this case, this evaluation was performed and the very short telomere length in both the lymphocytes and granulocytes, although not pathognomonic, seems to suggest a causative role for this variant, showing this analysis to be a valid tool to detect abnormalities in the shelterin components, as already described by Vulliamy et al. [31]. Moreover, the atypical presentation of this case, and the previously reported ones carrying such a variant, also suggest its correlation to a milder disease’s phenotype, which may depend on being close, but not inside, the amino acid hotspot, where most *TINF2* mutations are found, as already reported by Walne et al. [32]. Nonetheless, some cases with aplastic anemia carrying such a variant have been reported [21,32]. In fact, it is well known that telomeropathies are characterized by heterogeneous phenotypes, which may involve many organs, other than bone marrow, and may also become clearly manifest later in life. Moreover, in addition to the well-known variable expressivity of the disease, progressive telomere shortening has also been associated with disease anticipation and inter-generational heterogeneity of a clinical phenotype [33], which may also explain the absence of signs in the patient’s mother. Therefore, since telomere length analysis and molecular screening for telomeropathies are more likely to be performed in patients with bone marrow failure and other typical signs of the disease, other atypical cases might be underdiagnosed.

## 4. Conclusions

Our findings highlight the importance of genetic tests related to both marrow failure and immune-dysregulation diseases [25,34] as a means to identify specific disorders that may deserve targeted follow-up and treatment. In our case, given the absence of the characteristic stigmata of DC, we would have concluded a diagnosis of AIN and missed an early screening for BMF syndromes and specific follow-up, due to the disease-associated cancer predisposition. Indeed, recognizing a proper diagnosis of telomere biology disease, which implies a high susceptibility to toxicities from conventional therapies, is also crucial to avoid life-threatening events and to tailor treatments and conditioning regimens in case of stem cell transplantation [35,36,37,38,39,40].

## Figures and Tables

**Table 1:** Significant laboratory tests at admission in our Center.

	Results	Reference Range
Haemoglobin	13.3 g/dL	11.5–16.5
White blood cells	2080/mmc	4000–9800
Neutrophyls	150/mmc	2100–6430
Lymphocytes	1140/mmc	1000–2800
Platelets	148.000/mmc	150.000–450.000
Vitamin B12	816 pg/mL	191–663
IgA	201 mg/dL	70–400
IgM	67 mg/dL	40–230
IgG	1166 mg/dL	700–1600
IgG 1	696 mg/dL	370–1280
IgG 2	602 mg/dL	106–610
IgG 3	36.9 mg/dL	18–163
IgG 4	12.2 mg/dL	4–230
Folic acid	4.1 ng/mL	4.6–18.7

**Table 2:** Patient’s lymphocytes subsets.

	Results	Reference Range
CD3^+^	80.8% (921/mmc)	59–70%
CD3^+^CD4^+^	39%	37–50
CD3^+^CD8^+^	20.9%	20–27
CD3^+^HLA DR^+^	8.0%	4–6
CD19^+^	11.6% (132/mmc)	9–13%
CD16^+^CD56^+^CD3^-^	6.8% (78/mmc)	12–16%
CD16^+^CD56^+^CD3^+^	2.0%	3.3–4.6
CD4^+^/CD8^+^ ratio	1.9	1.5–2.2
CD25^+^	15.6%	NA
CD3^+^CD4^+^CD25br^+^CD45RA^-^	0.8%	0.6–0.8
CD3^+^CD45RA^+^	32.0%	24–33
CD3^+^CD45RO^+^	44.6%	36–49
CD3^+^TCR α/β^+^	63.7%	54–74
CD3^+^TCR γ/δ^+^	17.0%	3.3–4.6
CD3^+^TCR α/β^+^CD4^-^CD8^-^	3.9%	<1.5% of total lymphocytes
CD3^+^TCR α/β^+^CD4^-^CD8^-^ B220^+^ (%on total α/β^+^)	34.0%	<60.0
CD19^+^CD27^+^	21.5%	>15.0
CD3^+^CD25^+^/CD3^+^ HLA DR^+^ ratio	1.8	>1.0

**Table 3:** Coverage rate of all genes included in the next-generation sequencing (NGS) panel.

Target ID	Coverage Design	Mean Coverage Run	Target ID	Coverage Design	Mean Coverage Run
AP3B1	94.5	332.8	NRAS	100.0	369.6
CARD11	99.9	315.2	PIK3CD	98.4	274.9
CASP10	99.3	342.1	PIK3R1	99.7	445.1
CASP8	99.4	396.4	PRKCD	99.7	366.2
CD19	100.0	298.2	RAB27A	100.0	440.5
CD20	88.4	397.9	RAC2	100.0	288.2
CD40	100.0	413.2	RPL11	100.0	406.6
CD40LG	99.3	477.0	RPL26	100.0	437.0
CSF3R	100.0	351.8	RPL35A	100.0	471.8
CTC1 *	93.3	375.5	RPL5	100.0	370.8
CTLA4	100.0	374.0	RPS10	100.0	373.3
CXCR4	100.0	395.1	RPS17	99.1	142.3
DKC1 *	99.5	386.0	RPS19	100.0	318.8
ELANE	90.6	154.3	RPS24	97.9	383.8
FADD	92.8	236.5	RPS26	100.0	301.6
FAS	99.4	401.1	RPS7	100.0	375.3
FASLG	100.0	355.4	RTEL1 *	97.9	267.8
G6PC	99.0	410.6	SBDS	100.0	368.3
GFI1	97.5	218.6	SLC37A4	99.4	334.0
HAX1	100,0	426.9	TAZ	84.0	362.5
ITK	98,4	354.0	TERT *	88.7	214.9
JAGN1	100.0	354.0	TINF2 *	100.0	405.3
KRAS	100.0	403.7	TNFRSF13B	89.4	417.8
LAMTOR2	88.8	309.0	TNFRSF13C	33.9	304.5
LRBA	99.94	334.6	USB1	100.0	379.2
LYST	100.0	385.0	VPS13B	96.5	368.3
MAGT1	100,0	359.2	VPS45	96.7	370.7
NHP2 *	100,0	307.3	WAS	86.0	320.9
NOP10 *	100,0	407.0	WRAP53*	98.8	380.7

* genes related to telomeropathies.

**Table 4:** Colony forming unit (CFU) assay results.

	Results	Reference Range
CFU-E/BFU-E	35	27–81
CFU-GEMM	0	0–10
CFU-GM	33	33–100

Cells plated at 2 × 10^4^ in complete Methocult^TM^ GF H4434-SemCell Technologies and bone marrow progenitors scored after 14 days. CFU-E: Colony forming unit-erythroid/BFU-E: Burst forming unit-erythroid. CFU-GM: Colony forming unit-granulocyte, macrophage. CFU-GEMM: Colony forming unit-granulocyte, erythrocyte, macrophage, megakaryocyte.

## Data Availability

The original contributions presented in the study are included in the article; further inquiries can be directed to the corresponding author/s.
